# Association between Ménière’s disease and thyroid diseases: a nested case–control study

**DOI:** 10.1038/s41598-020-75404-y

**Published:** 2020-10-26

**Authors:** So Young Kim, Young Shin Song, Jee Hye Wee, Chanyang Min, Dae Myoung Yoo, Hyo Geun Choi

**Affiliations:** 1 Department of Otorhinolaryngology-Head and Neck Surgery, CHA Bundang Medical Center, CHA University, Seongnam, Korea; 2 Department of Internal Medicine, CHA Bundang Medical Center, CHA University, Seongnam, Korea; 3 Department of Otorhinolaryngology-Head & Neck Surgery, Hallym University College of Medicine, Anyang, Korea; 4 Hallym Data Science Laboratory, Hallym University College of Medicine, Anyang, Korea; 5 Graduate School of Public Health, Seoul National University, Seoul, Korea

**Keywords:** Endocrinology, Neurology

## Abstract

The association of thyroid disease and Ménière’s disease would suggest that both are autoimmune diseases. This study aimed to investigate the relation of goiter, hypothyroidism, thyroiditis, hyperthyroidism, and autoimmune thyroiditis with Ménière’s disease. The Korean National Health Insurance Service-Health Screening Cohort data from 2002 through 2015 were used. The 8183 adult patients with Ménière’s disease were 1:4 matched with the 32,732 individuals of the control group for age, sex, income, and region of residence. The previous histories of thyroid disorders including goiter, hypothyroidism, thyroiditis, and hyperthyroidism were investigated using conditional logistic regression analyses. Subgroup analyses were conducted, including for age and sex. Smoking, alcohol consumption, obesity, Charlson Comorbidity Index, histories of benign paroxysmal vertigo, vestibular neuronitis, other peripheral vertigo, thyroid cancer, and levothyroxine medication were adjusted in the models. The histories of goiter (5.7% vs. 4.2%), hypothyroidism (4.7% vs. 3.6%), thyroiditis (2.1% vs. 1.6%), hyperthyroidism (3.6% vs. 2.5%), and autoimmune thyroiditis (0.99% vs. 0.67%) were higher in the Meniere’s disease group than in the control group (all *P* < 0.05). The histories of goiter, hypothyroidism, and hyperthyroidism were associated with Ménière’s disease (adjusted odds ratio (OR) = 1.19 [95% confidence interval (CI) = 1.04–1.36] for goiter, 1.21 [95% CI 1.02–1.44] for hypothyroidism, and 1.27 [95% CI 1.09–1.49] for hyperthyroidism, each of *P* < 0.05). In subgroup analyses, hypothyroidism was associated with Ménière’s disease in < 65-year-old women. Hyperthyroidism was related with Ménière’s disease in women overall. Thyroid diseases of goiter, hypothyroidism, and hyperthyroidism were associated with Ménière’s disease.

## Introduction

The thyroid gland has crucial functions to regulate the endocrine systems through the hypothalamic-pituitary-thyroid axis and affect organ-specific or non-organ-specific metabolisms^1,2^. Therefore, metabolic syndrome, obesity, and type 2 diabetes have shown an association with thyroid diseases^3^. Autoimmune thyroid disease is thought to be associated with other organ-specific autoimmune diseases, such as Addison’s disease, type 1 diabetes mellitus, adrenocorticotropic hormone deficiency, and chronic active hepatitis, and non-organ-specific, such as rheumatoid arthritis, systemic sclerosis, and systemic lupus erythematosus^4^. Shared genetic predispositions and pathophysiology are thought to influence the association between autoimmune thyroid disease and many other autoimmune diseases^4,5^. The prevalence of autoimmune thyroid diseases, including Graves’ disease and chronic autoimmune thyroiditis or Hashimoto’s thyroiditis has been estimated about 1–5% in general population^6^. Hashimoto’s thyroiditis is the most common etiology of hypothyroidism (47%) and about 9.6% of hypothyroidism occurs due to the medication for a previous hyperthyroidism^7^. Although the abnormal thyroid function is primarily manifested as hyperthyroidism in Graves’ disease and hypothyroidism in Hashimoto's thyroiditis, both diseases shared common pathophysiology of genetic and epigenetic causes resulting in thyroid autoimmunity^8^. Thus, it could be theorized that both hyper- and hypo- thyroid function affect autoimmune diseases.

Ménière’s disease is clinically diagnosed by recurrent vertigo attacks combined with cochlear symptoms of primarily low- or mid-frequency sensorineural hearing loss, tinnitus, or ear fullness^9,10^. The incidence of Ménière’s disease varies according to the ethnic population, which is estimated to be about 13–200 person-years^11,12^. The peak age of onset of Ménière’s disease has been reported to be about 40–60 years^11,12^. The sudden surge of endolymphatic flow, which shifts from the pars inferior (cochlea) to the pars superior (utricle and semicircular canals), stimulates the vestibular hair cells in the cristae of the semicircular canals and may induce vertigo attack in patients with Ménière’s disease^13^. However, the pathophysiologic causes for the increase of endolymphatic flow are not understood and are thought to be multifactorial, including abnormal immune response and metabolic endocrine dysfunctions, such as hypothyroidism^13–15^. Previous studies have suggested the association of hypothyroidism with Ménière’s disease^14,16,17^. To our knowledge, there has been little research on thyroid diseases for a relationship with Ménière’s disease.

The hypothesis of the present study was that other thyroid diseases, besides hypothyroidism, might have an impact on the occurrence of Ménière’s disease, because of the autoimmunity and metabolic changes according to the abnormal thyroid function. To delineate the association of various thyroid diseases with Ménière’s disease, the histories of goiter, hypothyroidism, thyroiditis, and hyperthyroidism were compared between Ménière’s disease and control groups.

## Materials and methods

### Study population

The Ethics Committee of Hallym University (2019-10-023) approved this study. Requirement for written informed consent was waived by the Institutional Review Board of the Ethics Committee of Hallym University^18^. All analyses adhered to the guidelines and regulations of the ethics committee of Hallym University^18^. The detailed description of The Korean National Health Insurance Service-Health Screening Cohort data was described elsewhere^18^.

### Definition of Ménière’s disease (dependent variable)

If the participants were diagnosed with ICD-10 codes H810, we classified them as Ménière's disease^19^. From that group, we selected participants who were treated for Ménière's disease ≥ 2 times and had an audiometric examination (claim code: E6931–E6937, F6341–F6348) as a previous study^19^.

### Levothyroxine medications users (independent variable)

Levothyroxine medication users were selected if participants took levothyroxine medications for ≥ 3 months.

### Definition of Goiter (independent variable)

Goiter was defined if the participants were diagnosed with ICD-10 codes E04 (Other nontoxic goiter). Among them, we selected participants who were treated for goiter ≥ 2 times.

### Definition of hypothyroidism (independent variable)

Hypothyroidism was defined if the participants were diagnosed with ICD-10 codes E02 (Subclinical iodine-deficiency hypothyroidism) and E03 (Other hypothyroidism). Among them, we selected participants who underwent treatment ≥ 2 times.

### Definition of thyroiditis (independent variable)

Thyroiditis was defined if the participants were diagnosed with ICD-10 codes E06 (Thyroiditis). Among them, we selected the participants who treated for it ≥ 2 times.

### Definition of hyperthyroidism (independent variable)

Hyperthyroidism was defined if the participants were diagnosed with ICD-10 codes E05 (hyperthyroidism). Among them, we selected the participants who treated it ≥ 2 times.

### Definition of autoimmune thyroiditis (independent variable)

Autoimmune thyroiditis was defined if the participants were diagnosed with ICD-10 codes E063 (autoimmune thyroiditis). Among them, we selected the participants who were treated ≥ 2 times.

### Participant selection

**Figure 1:**
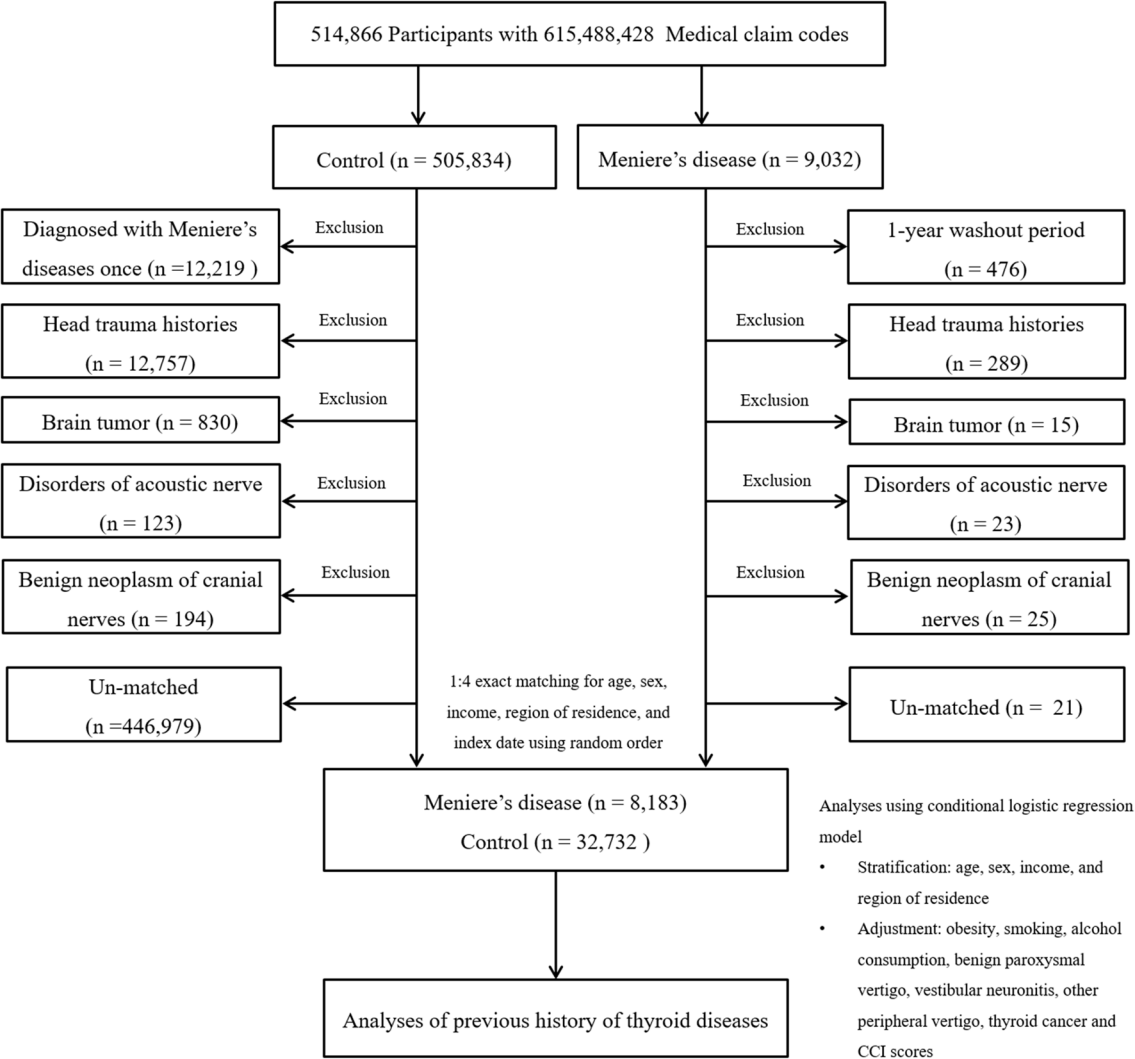
A schematic illustration of the participant selection process used in the present study. Of a total of 514,866 participants, 8,183 of Meniere’s disease patients were 1:4 matched with 32,732 control participants for age, sex, income, and region of residence.

Patients with Ménière’s disease were selected from 514,866 participants with 615,488,428 medical claim codes from 2002 through 2015 (n = 9032)^19^. The control group was included if participants were not diagnosed with Ménière’s disease from 2002 through 2015 (n = 505,834)^19^. To select a patient diagnosed with Ménière’s disease for the first time, patients diagnosed with Ménière’s disease in 2002 were excluded (washout periods, n = 476)^19^. Control participants were excluded if the participants were diagnosed with Ménière’s disease once (n = 12,219)^19^. Participants who were treated for head trauma (ICD-10 codes: S00 to S09, diagnosed by neurologists, neurosurgeons, or emergency medicine doctors) ≥ 2 times with head and neck CT evaluations (Claim codes: HA401–HA416, HA441–HA443, HA451–HA453, HA461–HA463, or HA471–HA473) were excluded (n = 289 patients with Ménière’s disease, n = 12,757 control participants)^19^. Participants who were treated for brain tumor (ICD-10 codes: C70 to C72) ≥ 2 times (n = 15 for Ménière’s disease, n = 830 for control), disorders of the acoustic nerve (ICD-10 codes: H933) ≥ 2 times (n = 23 for Ménière’s disease, n = 123 for control) and benign neoplasm of the cranial nerves (ICD-10 codes: D333) ≥ 2 times (n = 25 for Ménière’s disease, n = 194 for control) were excluded^19^. Patients with Ménière’s disease were 1:4 matched with control participants for age, sex, income, and region of residence^19^. To minimize the selection bias, the control participants were selected in a randomized order^18^. The index date of each patient with Ménière’s disease was set as the time of treatment of Ménière’s disease^19^. The index date of control participants was set as the index date of their matched patient with Ménière’s disease^19^. Therefore, each patient with Ménière’s disease matched with control participants had the same index date^19^. During the matching procedure, 21 patients with Ménière’s disease and 446,979 control participants were excluded^19^. Ultimately, 8,183 patients with Ménière’s disease were 1:4 matched with 32,732 control participants (Fig. 1)^19^.

### Covariates

Age groups were divided into 5-year intervals: 40–44, 45–49, 50–54, and 85+ years old (Total of 10 age groups)^20^. Income groups were classified as 5 classes (class 1 [lowest income]–5 [highest income]). The region of residence was grouped into urban and rural areas following our previous study^20^.

Tobacco smoking was categorized based on the participant’s current smoking status (nonsmoker, past smoker, and current smoker)^18^. Alcohol consumption was categorized based on the frequency of alcohol consumption (< 1 time a week and ≥ 1 time a week). Obesity was measured using body mass index (BMI, kg/m^2^)^18^. BMI was categorized as < 18.5 (underweight), ≥ 18.5 to < 23 (normal), ≥ 23 to < 25 (overweight), ≥ 25 to < 30 (obese I), and ≥ 30 (obese II) based on the Asia–Pacific criteria following the Western Pacific Regional Office (WPRO) 2000^21^. Missing BMI (23/43,290 [0.053%]) was replaced by mean values of variable from final selected participants.

The Charlson Comorbidity Index (CCI) has been used widely to measure disease burden using 17 comorbidities^22,23^. In our study, we excluded cancer and metastatic cancer from CCI score. CCI was measured as the continuous variable (0 [no comorbidities] through 29 [multiple comorbidities])^22,23^.

Regarding Ménière’s disease, benign paroxysmal vertigo (ICD-10 codes: H811), vestibular neuronitis (ICD-10 codes: H812), other peripheral vertigo (ICD-10 codes: H813), and thyroid cancer (ICD-10 codes: C73) were additionally assigned if participants were treated ≥ 2 times^19^.

### Statistical analyses

The general characteristics between the Ménière’s disease and control groups were compared using the Chi-square test^19^.

To analyze the odds ratios (ORs) with 95% confidence intervals (CIs), the conditional logistic regression model for Ménière’s disease in thyroid diseases was used^19^. The crude model 1 (adjusted for obesity, smoking, alcohol consumption, benign paroxysmal vertigo, vestibular neuronitis, other peripheral vertigo, thyroid cancer and CCI scores), and model 2 (additionally adjusted for Synthroid, goiter, hypothyroidism, thyroiditis, and hyperthyroidism in model 1) were used^19^. Because of levothyroxine medication, goiter, hypothyroidism, thyroiditis, and hyperthyroidism histories were closely related (S1 Table), we adjusted for them in model 2^19^. The analyses were stratified by age, sex, income, and region of residence^19^.

For the subgroup analyses, we divided participants by age and sex (< 65 years old and ≥ 65 years old; men and women) and by income and region of residence (low and high; urban and rural) using crude, model 1, and model 2^19^.

Two-tailed analyses were performed, and significance was defined as *P* values < 0.05. The SAS version 9.4 (SAS Institute Inc., Cary, NC, USA) were used for statistical analyses^19^.

## Results

The rates of goiter, hypothyroidism, thyroiditis, hyperthyroidism, and autoimmune thyroiditis were higher in the group with Ménière’s disease than in the control group (5.7% vs. 4.2% for goiter, 4.7% vs. 3.6% for hypothyroidism, 2.1% vs. 1.6% for thyroiditis, 3.6% vs. 2.5% for hyperthyroidism, and 0.99% vs. 0.67% for autoimmune thyroiditis, Table 1). The rate of levothyroxine medication was 4.8% in the Ménière’s disease group and 3.6% in the control group. Age, sex, income, and region of residence were exactly matched between the Ménière’s disease and control groups (*P* = 1.000). The distributions of obesity, smoking status, alcohol consumption, CCI score, benign paroxysmal vertigo, vestibular neuronitis, and other peripheral vertigo were significantly different between the Ménière’s disease and control groups (all *P* < 0.001).

**Table 1:** General characteristics of participants.

Characteristics	Total participants		
	Meniere’s disease (n, %)	Control (n, %)	*P* value
**Age (years old)**			1.000
40–44	123 (1.5)	492 (1.5)	
45–49	537 (6.6)	2148 (6.6)	
50–54	1199 (14.7)	4796 (14.7)	
55–59	1407 (17.2)	5628 (17.2)	
60–64	1349 (16.5)	5396 (16.5)	
65–69	1285 (15.7)	5140 (15.7)	
70–74	1151 (14.1)	4604 (14.1)	
75–79	736 (9.0)	2944 (9.0)	
80–84	316 (3.9)	1264 (3.9)	
85 +	80 (1.0)	320 (1.0)	
**Sex**			1.000
Male	2885 (35.3)	11,540 (35.3)	
Female	5298 (64.7)	21,192 (64.7)	
**Income**			1.000
1 (lowest)	1397 (17.1)	5588 (17.1)	
2	1038 (12.7)	4152 (12.7)	
3	1259 (15.4)	5036 (15.4)	
4	1707 (20.9)	6828 (20.9)	
5 (highest)	2782 (34.0)	11,128 (34.0)	
**Region of residence**			1.000
Urban	3445 (42.1)	13,780 (42.1)	
Rural	4738 (57.9)	18,952 (57.9)	
**Obesity**^**a**^			< 0.001*
Underweight	167 (2.0)	832 (2.5)	
Normal	2782 (34.0)	11,587 (35.4)	
Overweight	2290 (28.0)	8752 (26.7)	
Obese I	2694 (32.9)	10,442 (31.9)	
Obese II	250 (3.1)	1119 (3.4)	
**Smoking status**			
Nonsmoker	6640 (81.1)	25,851 (79.0)	< 0.001*
Past smoker	829 (10.1)	3017 (9.2)	
Current smoker	714 (8.7)	3864 (11.8)	
**Alcohol consumption**			
< 1 time a week	6209 (75.9)	23,867 (72.9)	< 0.001*
≥ 1 time a week	1974 (24.1)	8865 (27.1)	
**CCI score**			
0	5128 (62.7)	22,183 (67.8)	< 0.001*
1	1690 (20.7)	5447 (16.6)	
2	834 (10.2)	3071 (9.4)	
3	246 (3.0)	903 (2.8)	
≥ 4	285 (3.5)	1128 (3.5)	
Benign paroxysmal vertigo	2800 (34.2)	2170 (6.6)	< 0.001*
Vestibular neuronitis	900 (11.0)	467 (1.4)	< 0.001*
Other peripheral vertigo	1913 (23.4)	1517 (4.6)	< 0.001*
Thyroid cancer	81 (0.9)	296 (1.0)	0.469
**Period of taking levothyroxine**			
< 3 month	7793 (95.2)	31,557 (96.4)	< 0.001*
≥ 3 month	390 (4.8)	1175 (3.6)	
Goiter	462 (5.7)	1377 (4.2)	< 0.001*
Hypothyroidism	385 (4.7)	1168 (3.6)	< 0.001*
Thyroiditis	174 (2.1)	518 (1.6)	< 0.001*
Hyperthyroidism	292 (3.6)	805 (2.5)	< 0.001*
Autoimmune thyroiditis	81 (0.99)	219 (0.67)	0.002*

*CCI* Charlson comorbidity index.

*Chi-square test. Significance at *P* < 0.05.

^a^Obesity (BMI, body mass index, kg/m^2^) was categorized as < 18.5 (underweight), ≥ 18.5 to < 23 (normal), ≥ 23 to < 25 (overweight), ≥ 25 to < 30 (obese I), and ≥ 30 (obese II).

The histories of goiter, hypothyroidism, and hyperthyroidism were related with the increased OR for Ménière’s disease in model 2. The odds for Ménière’s disease were highest in hyperthyroidism, followed by hypothyroidism and goiter (adjusted OR 1.19, 95% CI 1.04–1.36 for goiter; adjusted OR 1.21, 95% CI 1.02–1.44 for hypothyroidism; adjusted OR 1.27, 95% CI 1.09–1.49 for hyperthyroidism, Table 2).

**Table 2:** Crude and adjusted odd ratios (95% confidence interval) for Meniere’s disease in levothyroxine, goiter, hypothyroidism, thyroiditis, hyperthyroidism, and autoimmune thyroiditis.

Characteristics	Odd ratios for Meniere’s disease					
	Crude^a^	*P* value	Model 1^a,b^	*P* value	Model 2^a,c^	*P* value
**Total participants (n = 40,915)**						
Levothyroxine	1.27 (1.12–1.44)	< 0.001*	1.28 (1.09–1.50)	0.003*	0.94 (0.76–1.16)	0.583
Goiter	1.37 (1.23–1.53)	< 0.001*	1.28 (1.13–1.45)	< 0.001*	1.19 (1.04–1.36)	0.011*
Hypothyroidism	1.34 (1.19–1.51)	< 0.001*	1.34 (1.17–1.53)	< 0.001*	1.21 (1.02–1.44)	0.030*
Thyroiditis	1.35 (1.14–1.61)	< 0.001*	1.23 (1.01–1.49)	0.041*	1.05 (0.85–1.29)	0.667
Hyperthyroidism	1.47 (1.28–1.69)	< 0.001*	1.37 (1.18–1.60)	< 0.001*	1.27 (1.09–1.49)	0.003*
Autoimmune thyroiditis	1.49 (1.15–1.92)	0.002*	1.26 (0.94–1.69)	0.122		

*CCI* Charlson Comorbidity Index.

*Conditional logistic regression model, significance at *P* < 0.05.

^a^Models stratified by age, sex, income, and region of residence.

^b^Model 1 was adjusted for obesity, smoking, alcohol consumption, benign paroxysmal vertigo, vestibular neuronitis, other peripheral vertigo, thyroid cancer, and CCI scores.

^c^Model 2 was adjusted for model 1 plus levothyroxine, goiter, hypothyroidism, thyroiditis, hyperthyroidism.

**Figure 2:**
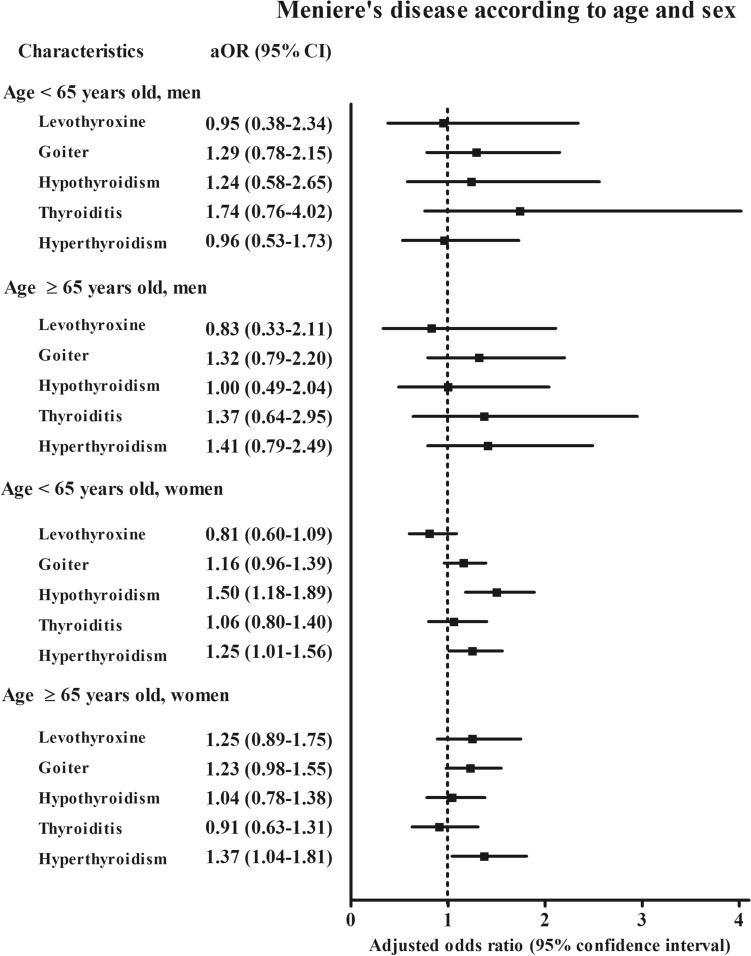
The odds ratios (95% confidence interval) of levothyroxine medication, goiter, hypothyroidism, thyroiditis, and hyperthyroidism for Meniere’s disease according to age and sex.

**Figure 3:**
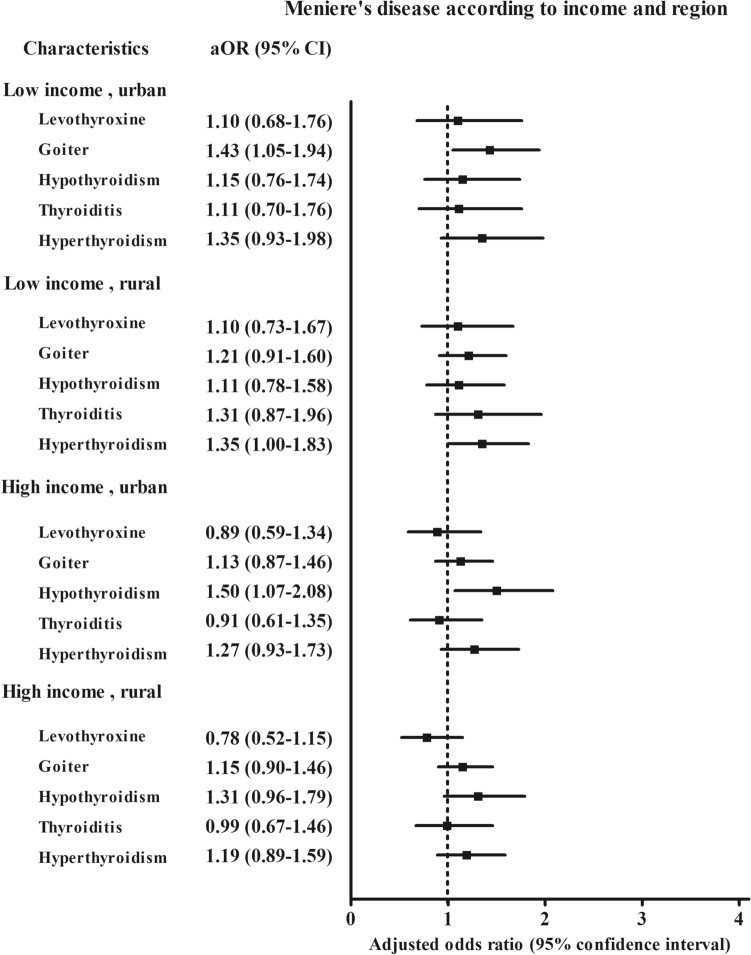
The odds ratios (95% confidence interval) of levothyroxine medication, goiter, hypothyroidism, thyroiditis, and hyperthyroidism for Meniere’s disease according to region of residence.

According to age and sex, hyperthyroidism was associated with the higher odds for Ménière’s disease in the women’s subgroups (adjusted OR 1.25, 95% CI 1.01–1.56 for the < 65-year-old women group and adjusted OR 1.37, 95% CI 1.04–1.81 for the ≥ 65-year-old women group) (Fig. 2 and S2 Table). Hypothyroidism was associated with a 1.5-fold higher odds for Ménière’s disease in the < 65-year-old women subgroup (95% CI 1.18–1.89). According to income and region of residence, goiter was 1.43 times more likely to be associated with Ménière’s disease in the low income, urban subgroup (95% CI 1.05–1.94, Fig. 3 and S3 Table). Hyperthyroidism was 1.35 times more likely associated with Ménière’s disease in the low income, rural subgroup (95% CI 1.00–1.98). Hypothyroidism was 1.5 times more likely to be associated with Ménière’s disease in the high income, urban subgroup (95% CI 1.07–2.08).

## Discussion

Ménière’s disease was positively related with the previous histories of goiter, hypothyroidism, and hyperthyroidism in the present study. Women showed a consistent association of Ménière’s disease with hypothyroidism (< 65 years old) and hyperthyroidism. This study adjusted for levothyroxine medication and other thyroid diseases, thereby separately analyzing the relation of each thyroid disease with Ménière’s disease.

Previous studies described the relation of hypothyroidism with Ménière’s disease^14,17^. However, most prior studies were limited with a small sample size^17,24^. In a case–control study, the rate of thyroid hormone medication was higher in patients with Ménière’s disease than in the age- and sex-matched control group (32% [16/50] vs. 4% [2/50], *P* < 0.001)^17^. In another small case series, the thyroxin medication improved the symptoms of Ménière’s disease patients with hypothyroidism (100%, 12/12)^24^. A cohort study conducted among 27,050 individuals in Taiwan demonstrated a 1.31-fold higher odds for hypothyroidism in patients with Ménière’s disease (95% CI 1.14–1.51)^16^. However, they did not consider other thyroid diseases.

The inflammatory or metabolic changes in the patients with thyroid diseases could have an impact on the inner ear inflammation and homeostasis of endolymphatic flow. The association of inflammation with thyroid dysfunction has been acknowledged^25^. For instance, inflammatory cytokines including tumor necrosis factor α and interleukin 1 and 6 reduced the expressions of sodium/iodine symporters, in that impeded the iodide uptake in the thyroid gland^25,26^. Moreover, the association of thyroid diseases with the metabolic disease of obesity has been reported based on the inflammatory and metabolic etiology^27,28^. The inflammatory or degenerative changes of the inner ear epithelia could increase the risk of Ménière’s disease. Although the contribution of autoimmune response to the pathogenesis of Ménière’s disease has been suggested, only the immune complexes were observed only in about 7% of patients with Ménière’s disease and the biomarker for autoimmunity in Ménière’s disease is still elusive^29^. A few endotypes of Ménière’s diseases were suggested with different etiologies inducing hypoplasia or inflammation or degeneration of the endolymphatic sac^30,31^. The maintenance of ionic and non-ionic compositions of endolymph is crucial for transduction of acceleration in the vestibular labyrinth, which consists of a unique composition of low calcium levels of endolymph (280 μM) compared to perilymph (1 Mm)^32,33^. Thus, the perturbation of this composition due to altered metabolism could impact the vestibular function. Indeed, it was suggested that the abnormal metabolisms of patients with thyroid disease could induce the endolymphatic hydrops and Ménière’s disease^14,15^. Hypothyroidism supposedly changes the composition of endolymphatic fluid through the diffusion of thyroid autoantibody complexes in the endolymph^34^. In addition, the common anion exchanger such as pendrin, which is encoded by Solute Carrier 26A4 (SLC26A4), could be defected in the patients with goiter and/or hypothyroidism. Therefore, altering the endolymph composition and endocochlear potential, as in the case of Pendred syndrome^35^.

Although autoimmune thyroiditis did not show a statistically significant association with Ménière’s disease in this study, the abnormal autoimmune responses in the patients with autoimmune thyroid disease could influence the inner ear autoimmune dysfunctions related with Ménière’s disease^36–38^. Patients with Ménière’s disease had a higher rate of thyroid autoantibodies of anti-thyroperoxidase antibody, anti-TSH receptor antibody, anti-thyroperoxidase antibody, anti-thyroglobulin antibody compared to control and acute unilateral peripheral vestibulopathy groups^38^. Ménière’s disease, especially in bilateral cases, has been reported to be related with immune dysfunctions including allergy^36,37,39,40^. A prospective case–control study suggested the higher rate of positivity to cellular and humoral autoimmune tests in 10 bilateral patients with Ménière’s disease^36^. In addition, the prevalence of systemic autoimmune diseases, such as rheumatoid arthritis, systemic lupus erythematosus, and ankylosing spondylitis was higher in 690 patients with Ménière’s disease than in the general population^41^. In particular, familial cases of Ménière’s disease have been reported to harbor some genetic factors, such as certain human leucocyte antigens^29,42^. Therefore, it was presumed that Ménière’s disease could be included in the spectrum of autoimmune inner ear disease^41^. Autoimmune mechanism may have an influence on hypothyroidism, hyperthyroidism, and thyroiditis^8^. Therefore, the possible mediating role of autoimmunity with respect to thyroid diseases in Ménière’s disease cannot be excluded in this study.

Both hypo- and hyperthyroidism were related to Ménière’s disease in this study. These associations could have originated from the common pathophysiology among the thyroid diseases, such as autoimmune responses and inflammation^4,5^. In addition, the effects of treatment of abnormal thyroid function could influence the overlapping of thyroid diseases. For instance, the treatment of hypothyroidism could induce a status of hyperthyroidism. Thus, it was reported that the treatment of hypothyroidism accounted for approximately 9.6% of causes of hypothyroidism^7^. Moreover, it was reported that about 14% of patients with Ménière’s disease showed a state of hyperthyroidism due to l-thyroxine therapy^38^.

The present study was based on a large, representative national cohort. The large study population guaranteed a sufficient number of control population matched for age, sex, income, and region of residence. In addition, potential confounders were comprehensively reviewed and adjusted for obesity: smoking; alcohol consumption; comorbidities using CCI score; other vestibular diseases of benign paroxysmal positional vertigo, vestibular neuronitis, and other peripheral vertigo; levothyroxine medication; and other thyroid diseases of goiter, hypothyroidism, thyroiditis, and hyperthyroidism. The use of the health claim data meant that the thyroid status could not be measured by thyroid function tests because there was a possibility that the thyroid status was heterogenous among participants. In addition, subclinical or untreated thyroid diseases could be misclassified. For Ménière’s disease, the vestibular function tests could not be assessed; thus the severity and management of Ménière’s disease might vary among participants. However, the correlation of vestibular function tests with the types and severity of Ménière’s disease could also be variable, and the clinical otovestibular symptoms assessed by the otologist might be more reliable for the diagnosis of Ménière’s disease. The endotypes of Ménière’s disease, including a degenerating distal endolymphatic sac and hypoplastic endolymphatic sac, could not be differentiated in the present study^31^. Although we could not access low- to-medium frequency sensorineural hearing loss, which is accompanied in the definite Ménière’s disease, the participants who underwent pure tone audiometry were enrolled, thus, probable Ménière’s disease might be included in this study^43^.

## Conclusion

Thyroid diseases of goiter, hypothyroidism, and hyperthyroidism were related with the increased risk of Ménière’s disease. The associations of hypo- and hyperthyroidism with Ménière’s disease were consistent in women.

## Supplementary information

Supplementary Tables.

## Data Availability

Releasing of the data by the researcher is not allowed legally. All data are available from the database of the National health Insurance Sharing Service (NHISS) https://nhiss.nhis.or.kr/. NHISS allows data access, at a particular cost, for any researcher who promises to follow the research ethics. Data of this article can be downloaded from the website after promising to follow the research ethics.
