# Supplementary material for: Association between Ménière’s disease and thyroid diseases: a nested case–control study
Source: Sci Rep. 2020 Oct 26;10:18224. doi: 10.1038/s41598-020-75404-y (PMC7588449; doi:10.1038/s41598-020-75404-y)
Supplement: Supplementary file 1 — Supplementary Tables. [file 41598_2020_75404_MOESM1_ESM.docx]

**Association between Ménière’s disease and thyroid diseases: A nested case-control study**

So Young Kim, MD, PhD^1^, Young Shine Song, MD, PhD^2^, Jee Hye Wee, MD, PhD ^3^, Chanyang Min, PhD^4,5^, Dae Myoung Yoo, MS^4^, Hyo Geun Choi, MD, PhD ^3,4*^

^1^Department of Otorhinolaryngology-Head & Neck Surgery, CHA Bundang Medical Center, CHA University, Seongnam, Korea

^2^Department of Internal Medicine, CHA Bundang Medical Center, CHA University, Seongnam, Korea

^3^Department of Otorhinolaryngology-Head & Neck Surgery, Hallym University College of Medicine, Anyang, Korea

^4^Hallym Data Science Laboratory, Hallym University College of Medicine, Anyang, Korea

^5^Graduate School of Public Health, Seoul National University, Seoul, Korea

**Running title**: Ménière’s disease and thyroid diseases

***Correspondence:** Hyo Geun Choi [pupen@naver.com](mailto:pupen@naver.com)

**S1 Table** Pearson’s chi-square test between each of levothyroxine, goiter, hypothyroidism, thyroiditis, hyperthyroidism, and autoimmune thyroiditis

|  |  | Levothyroxine | Goiter | Hypothyroidism | Thyroiditis | Hyperthyroidism | Autoimmune  thyroiditis |
| --- | --- | --- | --- | --- | --- | --- | --- |
| Levothyroxine | χ² | 1 |  |  |  |  |  |
|  | P-value |  |  |  |  |  |  |
| Goiter | χ² | 5876.0 | 1 |  |  |  |  |
|  | P-value | <0.001* |  |  |  |  |  |
| Hypothyroidism | χ² | 16815.7 | 2218.4 | 1 |  |  |  |
|  | P-value | <0.001* | <0.001* |  |  |  |  |
| Thyroiditis | χ² | 2945.0 | 1300.7 | 2087.7 | 1 |  |  |
|  | P-value | <0.001* | <0.001* | <0.001* |  |  |  |
| Hyperthyroidism | χ² | 1670.3 | 810.2 | 1268.3 | 687.2 | 1 |  |
|  | P-value | <0.001* | <0.001* | <0.001* | <0.001* |  |  |
| Autoimmune thyroiditis | χ² | 2353.1 | 904.3 | 1870.3 | 17566.6 | 576.9 | 1 |
|  | P-value | <0.001* | <0.001* | <0.001* | <0.001* | <0.001* |  |

* Chi-square test. Significance at P < 0.05

**S2 Table** Subgroup analyses of crude and adjusted odd ratios (95% confidence interval) for Meniere’s disease in levothyroxine, goiter, hypothyroidism, thyroiditis, hyperthyroidism, and autoimmune thyroiditis according to age and sex

| Characteristics | | Odd ratios for Meniere’s disease | | | | | |
| --- | --- | --- | --- | --- | --- | --- | --- |
|  |  | Crude† | P-value | Model 1†‡ | P-value | Model 2†§ | P-value |
| Age < 65 years old, men (n= 8,000) | | | | | | | |
|  | Levothyroxine | 1.13 (0.64-2.02) | 0.670 | 1.27 (0.60-2.69) | 0.533 | 0.95 (0.38-2.34) | 0.906 |
|  | Goiter | 1.42 (0.93-2.16) | 0.102 | 1.30 (0.68-2.49) | 0.435 | 1.29 (0.78-2.15) | 0.325 |
|  | Hypothyroidism | 1.13 (0.64-2.02) | 0.670 | 1.30 (0.68-2.49) | 0.435 | 1.24 (0.58-2.65) | 0.580 |
|  | Thyroiditis | 1.92 (0.93-3.94) | 0.828 | 1.84 (0.82-4.13) | 0.870 | 1.74 (0.76-4.02) | 0.192 |
|  | Hyperthyroidism | 1.06 (0.63-1.79) | 0.828 | 1.05 (0.59-1.88) | 0.870 | 0.96 (0.53-1.73) | 0.886 |
|  | Autoimmune thyroiditis | 1.78 (0.55-5.79) | 0.338 | 1.61 (0.44-5.85) | 0.473 |  |  |
| Age ≥ 65 years old, men (n= 6,425) | | | | | | | |
|  | Levothyroxine | 0.89 (0.50-1.59) | 0.688 | 1.02 (0.51-2.04) | 0.958 | 0.83 (0.33-2.11) | 0.701 |
|  | Goiter | 1.31 (0.83-2.06) | 0.252 | 1.35 (0.82-2.23) | 0.243 | 1.32 (0.79-2.20) | 0.293 |
|  | Hypothyroidism | 1.09 (0.68-1.75) | 0.728 | 1.04 (0.60-1.78) | 0.900 | 1.00 (0.49-2.04) | 0.996 |
|  | Thyroiditis | 1.63 (0.85-3.11) | 0.139 | 1.36 (0.64-2.89) | 0.418 | 1.37 (0.64-2.95) | 0.416 |
|  | Hyperthyroidism | 1.34 (0.80-2.23) | 0.263 | 1.41 (0.81-2.46) | 0.226 | 1.41 (0.79-2.49) | 0.243 |
|  | Autoimmune thyroiditis | 3.51 (1.27-9.67) | 0.016* | 2.89 (0.89-9.35) | 0.077 |  |  |
| Age < 65 years old, women (n= 15,075) | | | | | | | |
|  | Levothyroxine | 1.14 (0.95-1.36) | 0.149 | 1.22 (0.98-1.53) | 0.082 | 0.81 (0.60-1.09) | 0.167 |
|  | Goiter | 1.29 (1.11-1.50) | <0.001* | 1.22 (1.03-1.45) | 0.024* | 1.16 (0.96-1.39) | 0.121 |
|  | Hypothyroidism | 1.38 (1.17-1.62) | <0.001* | 1.45 (1.20-1.74) | <0.001* | 1.50 (1.18-1.89) | <0.001* |
|  | Thyroiditis | 1.36 (1.08-1.70) | 0.009* | 1.23 (0.95-1.59) | 0.125 | 1.06 (0.80-1.40) | 0.689 |
|  | Hyperthyroidism | 1.48 (1.23-1.78) | <0.001* | 1.35 (1.09-1.67) | 0.006* | 1.25 (1.01-1.56) | 0.041* |
|  | Autoimmune thyroiditis | 1.36 (0.98-1.88) | 0.066 | 1.06 (0.72-1.55) | 0.772 |  |  |
| Age ≥ 65 years old, women (n= 11,415) | | | | | | | |
|  | Levothyroxine | 1.59 (1.29-1.95) | <0.001* | 1.44 (1.13-1.85) | 0.004* | 1.25 (0.89-1.75) | 0.194 |
|  | Goiter | 1.52 (1.26-1.84) | <0.001* | 1.36 (1.10-1.68) | 0.005* | 1.23 (0.98-1.55) | 0.071 |
|  | Hypothyroidism | 1.36 (1.11-1.66) | 0.003* | 1.27 (1.02-1.59) | 0.033* | 1.04 (0.78-1.38) | 0.803 |
|  | Thyroiditis | 1.22 (0.88-1.68) | 0.238 | 1.10 (0.77-1.56) | 0.604 | 0.91 (0.63-1.31) | 0.606 |
|  | Hyperthyroidism | 1.62 (1.27-2.06) | <0.001* | 1.49 (1.14-1.94) | 0.004* | 1.37 (1.04-1.81) | 0.024* |
|  | Autoimmune thyroiditis | 1.48 (0.90-2.45) | 0.127 | 1.42 (0.82-2.46) | 0.214 |  |  |

Abbreviation: CCI, Charlson Comorbidity Index

* Conditional logistic regression model, Significance at P < 0.05

† Models stratified by age, sex, income, and region of residence.

‡ Model 1 was adjusted for obesity, smoking, alcohol consumption, benign paroxysmal vertigo, vestibular neuronitis, other peripheral vertigo, thyroid cancer, and CCI scores.

§ Model 2 was adjusted for model 1 plus levothyroxine, goiter, hypothyroidism, thyroiditis, and hyperthyroidism.

**S3 Table** Subgroup analyses of crude and adjusted odd ratios (95% confidence interval) for Meniere’s disease in levothyroxine, goiter, hypothyroidism, thyroiditis, hyperthyroidism, and autoimmune thyroiditis according to income and region

| Characteristics | | Odd ratios for Meniere’s disease | | | | | |
| --- | --- | --- | --- | --- | --- | --- | --- |
|  |  | Crude† | P-value | Model 1†‡ | P-value | Model 2†§ | P-value |
| Low income , urban (n= 7,230) | | | | | | | |
|  | Levothyroxine | 1.49 (1.12-1.98) | 0.007* | 1.49 (1.03-2.14) | 0.033* | 1.10 (0.68-1.76) | 0.702 |
|  | Goiter | 1.65 (1.29-2.11) | <0.001* | 1.55 (1.16-2.07) | 0.003* | 1.43 (1.05-1.94) | 0.023* |
|  | Hypothyroidism | 1.36 (1.02-1.81) | 0.038* | 1.37 (0.99-1.90) | 0.061 | 1.15 (0.76-1.74) | 0.507 |
|  | Thyroiditis | 1.50 (1.02-2.20) | 0.039* | 1.41 (0.92-2.17) | 0.118 | 1.11 (0.70-1.76) | 0.644 |
|  | Hyperthyroidism | 1.53 (1.11-2.12) | 0.010* | 1.52 (1.05-2.19) | 0.026* | 1.35 (0.93-1.98) | 0.118 |
|  | Autoimmune thyroiditis | 1.96 (1.09-3.51) | 0.024* | 1.47 (0.75-2.89) | 0.264 |  |  |
| Low income , rural (n= 11,240) | | | | | | | |
|  | Levothyroxine | 1.45 (1.13-1.85) | 0.004* | 1.43 (1.05-1.95) | 0.024* | 1.10 (0.73-1.67) | 0.649 |
|  | Goiter | 1.47 (1.17-1.85) | <0.001* | 1.36 (1.04-1.76) | 0.022* | 1.21 (0.91-1.60) | 0.190 |
|  | Hypothyroidism | 1.31 (1.03-1.67) | 0.025* | 1.33 (1.02-1.75) | 0.038* | 1.11 (0.78-1.58) | 0.555 |
|  | Thyroiditis | 1.64 (1.17-2.31) | 0.004* | 1.53 (1.04-2.25) | 0.031* | 1.31 (0.87-1.96) | 0.192 |
|  | Hyperthyroidism | 1.66 (1.28-2.15) | <0.001* | 1.48 (1.10-1.98) | 0.009* | 1.35 (1.00-1.83) | 0.049* |
|  | Autoimmune thyroiditis | 2.22 (1.31-3.74) | 0.003* | 1.76 (0.95-3.24) | 0.072 |  |  |
| High income , urban (n= 9,995) | | | | | | | |
|  | Levothyroxine | 1.10 (0.86-1.41) | 0.468 | 1.27 (0.94-1.73) | 0.127 | 0.89 (0.59-1.34) | 0.570 |
|  | Goiter | 1.23 (1.00-1.52) | 0.053 | 1.19 (0.94-1.52) | 0.152 | 1.13 (0.87-1.46) | 0.356 |
|  | Hypothyroidism | 1.42 (1.14-1.78) | 0.002* | 1.45 (1.13-1.88) | 0.004* | 1.50 (1.07-2.08) | 0.017* |
|  | Thyroiditis | 1.17 (0.84-1.65) | 0.351 | 1.05 (0.72-1.54) | 0.801 | 0.91 (0.61-1.35) | 0.625 |
|  | Hyperthyroidism | 1.46 (1.12-1.92) | 0.006* | 1.34 (0.98-1.82) | 0.633 | 1.27 (0.93-1.73) | 0.139 |
|  | Autoimmune thyroiditis | 1.46 (0.95-2.24) | 0.224 | 1.35 (0.83-2.18) | 0.224 |  |  |
| High income , rural (n= 12,450) | | | | | | | |
|  | Levothyroxine | 1.18 (0.92-1.50) | 0.189 | 1.04 (0.76-1.41) | 0.823 | 0.78 (0.52-1.15) | 0.211 |
|  | Goiter | 1.28 (1.05-1.56) | 0.015* | 1.17 (0.93-1.46) | 0.184 | 1.15 (0.90-1.46) | 0.258 |
|  | Hypothyroidism | 1.28 (1.03-1.59) | 0.027* | 1.23 (0.96-1.57) | 0.101 | 1.31 (0.96-1.79) | 0.084 |
|  | Thyroiditis | 1.21 (0.86-1.70) | 0.272 | 1.53 (1.04-2.25) | 0.031* | 0.99 (0.67-1.46) | 0.957 |
|  | Hyperthyroidism | 1.29 (1.00-1.67) | 0.047* | 1.23 (0.93-1.63) | 0.143 | 1.19 (0.89-1.59) | 0.241 |
|  | Autoimmune thyroiditis | 0.81 (0.45-1.48) | 0.492 | 0.71 (0.36-1.39) | 0.320 |  |  |

Abbreviation: CCI, Charlson Comorbidity Index

* Conditional logistic regression model, Significance at P < 0.05

† Models stratified by age, sex, income, and region of residence.

‡ Model 1 was adjusted for obesity, smoking, alcohol consumption, benign paroxysmal vertigo, vestibular neuronitis, other peripheral vertigo, thyroid cancer, and CCI scores.

§ Model 2 was adjusted for model 1 with levothyroxine, goiter, hypothyroidism, thyroiditis, and hyperthyroidism.
